# Qualitative phytochemical profiling, antioxidant activity, and development of a water-in-oil cream containing combined oil and water infusions of frankincense resin (*Boswellia spp.*): a preliminary *in vitro* study

**DOI:** 10.3389/jpps.2026.15609

**Published:** 2026-04-28

**Authors:** Shamama Javed, Ahmad Salawi, Sivakumar S. Moni, Waquar Ahsan, Gulrana Khuwaja, Md Shamsher Alam, Durgaramani Sivadasan, Aamena Jabeen, Maram Yahya Aziabi, Hind Mohammed Suwaydi, Taif Eassa M. Alajam, Amwaj Yahya Marwai Nammazi, Nourah Mohammed Ahmed Kadumi

**Affiliations:** 1 Department of Pharmaceutics, College of Pharmacy, Jazan University, Jazan, Saudi Arabia; 2 Health Research Centre, Jazan University, Jazan, Saudi Arabia; 3 Department of Pharmaceutical Chemistry and Pharmacognosy, College of Pharmacy, Jazan University, Jazan, Saudi Arabia; 4 College of Pharmacy, Jazan University, Jazan, Saudi Arabia

**Keywords:** antioxidant, *Boswellia*, boswellic acids, frankincense, gum olibanum

## Abstract

**Background:**

Frankincense (gum olibanum, *Boswellia* spp.) is an oleo-gum resin widely used in traditional medicine and cosmetics owing to the presence of volatile oils and pentacyclic triterpenic acids (boswellic acids) with reported anti-inflammatory and antioxidant properties.

**Objectives:**

The aim of this study was to extract and characterize oil- and water-soluble fractions of frankincense resin, evaluate their antioxidant potential, and incorporate them into a stable water-in-oil (*w/o*) nourishing/antiaging cream.

**Methods:**

Frankincense resin was ground and macerated separately in sweet almond oil and Madinah rosewater to yield oil and water infusions, respectively. Qualitative phytochemical tests and FT-IR spectroscopy were employed for the characterization of both extracts and final formulations. Antioxidant potential was assessed using the DPPH assay followed by the development of a *w/o* cream (beeswax:almond oil:rosewater base) using combined infusions, which was evaluated for pH, viscosity, phase separation, spreadability, and thermal stability.

**Results:**

Phytochemical screening showed presence of triterpenoids and boswellic type functionalities predominantly in the oil infusion, whereas saponins and minor alkaloids were detected in the water infusion. In the DPPH assay, significant free radical scavenging activity was observed as sample 2 showed 71% inhibition at 343.46 ± 34.2 μg/mL. The developed cream formulation showed good physical stability, acceptable pH and shear-thinning rheology.

**Conclusion:**

A stable, all-natural *w/o* cream formulation was developed incorporating combined oil- and water-soluble frankincense infusions. Future studies are warranted to perform quantitative chemical analysis, *in vitro* skin permeation, and formal skin safety testing to ensure uninform active content, good bioavailability and tolerability prior to clinical studies.

## Introduction

From time immemorial, plants and their derived products have played a fundamental role in meeting human needs, providing food, fragrances, flavors, dyes, and medicinal agents. Among the diverse array of plant-derived materials, aromatic resins have occupied an important position due to their multifaceted uses in medicine, perfumery, cosmetics and cultural rituals. One such resin, frankincense, also known as the gum olibanum, has been a valuable commodity for commerce and continues to be an important raw material in both traditional and modern applications [1–6]. Frankincense is a natural aromatic oleo-gum resin exuded from the bark of various species of the genus *Boswellia* (family Burseraceae). There are 25 species in the genus *Boswellia*. These species can be found in many places, including North Africa, Somalia (*B*. *carterii* and *B*. *frereana*), Ethiopia (*B*. *papyrifera* and *B*. *rivae*), India (*B*. *serrata*), the Arabian Peninsula (*B*. *sacra*), and Ethiopia (*B*. *neglecta*) [7, 8]. It is primarily obtained from making deliberate incisions into the bark of mature trees leading to a fragrant exudate to seep out and subsequently harden to afford tear-shaped resin droplets. The resin is composed mainly of three fractions: volatile oil, alcohol-soluble resin, and water-soluble gum. The volatile fraction is responsible for its characteristic aroma, while the resin fraction is rich in pentacyclic triterpenic acids, also referred to as boswellic acids, responsible for majority of its therapeutic properties. On the contrary, the gum fraction consists mainly of polysaccharides and water-soluble oligosaccharides [9].

**FIGURE 1:**
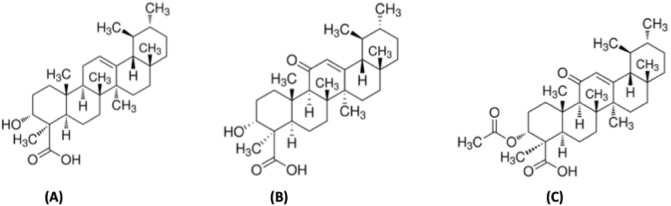
Chemical structures of the major triterpenoids present in frankincense resin (*Boswellia* sp.). **(A)** β-boswellic acid; **(B)** 11-keto-β-boswellic acid; and **(C)** acetyl-11-keto-β-boswellic acid.

Traditionally, frankincense has been widely employed in incense burning, religious rituals, and perfumery along with its use in alternative medicine systems, including Ayurveda, Traditional Chinese Medicine, and Arabian herbal practices. Its therapeutic potential is associated with a variety of biological activities such as antimicrobial, antioxidant, anti-inflammatory, and anti-proliferative properties [10–13]. The boswellic acids present in the resin includes β-boswellic acid, 11-keto-β-boswellic acid (KBA), and acetyl-11-keto-β-boswellic acid (AKBA) (Figure 1). These acids are pentacyclic triterpenoids and have been reported to modulate inflammatory pathways by inhibiting the activity of pro-inflammatory enzymes, including 5-lipoxygenase and human leukocyte elastase [14]. The volatile fraction of frankincense contains a complex mixture of monoterpenes, diterpenes, and sesquiterpenes, contributing to its aroma and pharmacological effects.

The dermatological application of frankincense is supported by several mechanisms of action including new cell generation, enhancement of skin elasticity, reduction of depth and appearance of wrinkles, and by soothing dry and irritated skin [15]. These effects are results of its combined anti-inflammatory and antioxidant actions in addition to the ability of lipophilic terpenoids to penetrate stratum corneum resulting in the modulation of dermal processes. Moreover, frankincense extract is known to mitigate oxidative stress, a major contributor to skin aging, and therefore it may offer protection against photo-aging and environment-related skin damage. In a previous study, frankincense was reported to lower levels of intracellular cell adhesion molecule 1 (ICAM1) and interferon gamma-induced protein 10 (IP-10) *in vitro*, two significant inflammatory indicators causing unexpected skin sensitivity [16]. Frankincense-containing formula could alleviate itching and erythema in individuals with erythematous eczema and psoriasis, as evidenced by *in vivo* experiments confirming its anti-inflammatory action [17]. The gum resin extracted from *B. serrata* was utilized for a number of skin conditions. Through the TLR7/8 route, acetyl-11-keto-β-boswellic acid prevented dendritic cells from secreting cytokines *in vitro* and in a mouse model of imiquimod-induced psoriasis [18]. In extremely metastatic melanoma, boswellic acid acetate induced apoptosis and differentiation [19, 20].

From a formulation perspective, frankincense resin presents a unique opportunity owing to the complementary bioactive components present in both its water- and oil-soluble fractions. The water-soluble fraction is rich in polysaccharides which can contribute to skin hydration and support the skin barrier, while the oil-soluble fraction is rich in triterpenoids and essential oils which is well-suited for lipid-based delivery systems. Incorporating both fractions in a single topical formulation would lead to synergistic effects, and thereby could maximize the therapeutic potential of the resin. A water-in-oil (*w/o*) emulsion system is particularly suitable for such application as it can efficiently incorporate both lipophilic and hydrophilic components, providing an occlusive effect to reduce trans-epidermal water loss and enhance the stability of sensitive active ingredients [21].

Given these considerations, the present study was designed to prepare oil and water infusions of locally procured frankincense resin separately, characterize, and evaluate them for their antioxidant potential. This was followed by the development of *w/o* nourishing and anti-aging cream by incorporating both oil- and water-soluble infusions. Eventually, the physical, chemical, and stability parameters of the final formulation was evaluated to determine its suitability for cosmetic applications. By combining traditional folklore and modern analytical formulation techniques, this work was aimed to provide a scientific basis for the topical application of frankincense resin, potentially contributing to the development of effective and all-natural anti-aging skincare product.

## Materials and methods

### Materials

Frankincense resin (gum olibanum) was procured locally from Elhekma company, Jazan, Saudi Arabia. Sweet almond oil (NOW® Solutions, Bloomingdale, IL, USA), used as lipophilic extraction medium, was purchased from a herbal pharmacy, while Madinah rosewater (Nada Rabee company, Madinah, Saudi Arabia) was obtained from a commercial supplier in Madinah, Saudi Arabia. Beeswax and other standard laboratory reagents were of analytical grade and were purchased from Sigma Aldrich (Steinheim, Germany). FT-IR Spectra were recorded using a SHIMADZU-IRSpirit-T spectrophotometer (Kyoto, Japan), while the UV-Vis spectrophotometric readings were recorded using a Cary 100 Bio UV-Vis spectrophotometer (Agilent Technologies, USA).

### Preparation of frankincense infusions

The frankincense resin was first ground into fine powder using a household coffee grinder to increase the surface area for extraction. The powdered resin was then divided into two equal portions for the preparation of oil- and water-soluble infusions.

#### Oil infusion

10 g of finely powdered resin was suspended to 40 g of sweet almond oil. The mixture was heated to 75 °C for 1 h with occasional stirring to facilitate dissolution and then allowed to stand at room temperature for 3 days to allow maximum extraction of the lipophilic constituents. After the extraction period, mixture was filtered through muslin cloth to remove undissolved material, yielding the oil infusion of frankincense.

#### Water infusion

A separate 10 g portion of powdered resin was suspended in 40 g of Madinah rosewater and similar to the oil-infusion, the mixture was warmed to 75 °C for 1 h and then left to stand at room temperature for 3 days. After the maceration process is over, the infusion was filtered to remove undissolved particles, yielding the water infusion.

Both the prepared infusions were stored in amber-colored bottles under refrigerated conditions until further use in phytochemical analysis, antioxidant assays, and formulation development.

### Qualitative phytochemical analyses

The oil and water infusions were subjected to standard qualitative chemical tests to preliminarily identify the major phytoconstituents of frankincense resin, particularly triterpenoids such as boswellic acids, along with other resinous and volatile components. Common chemical tests were employed in this study to determine authenticity and chemical nature of the resin. The intensity of reactions was recorded qualitatively as negative (−), trace (+/−), present (+), moderately present (++), or strongly present (+++).

#### Salkowski test (for triterpenoids and steroids)

A small amount of the resin infusion was dissolved in chloroform. A few drops of concentrated sulfuric acid were carefully added along the side of the test tube. A reddish-brown or golden-yellow color at the interface indicated the presence of triterpenoids or steroids [22].

#### Liebermann-Burchard test (for triterpenoids)

The infusions were dissolved in acetic anhydride. A drop or two of concentrated sulfuric acid were added slowly. A color change from pink to purple or blue-green indicated the presence of triterpenoids or sterols [23].

#### Acetylation test (for hydroxyl groups in boswellic acids)

The resin infusion was treated with acetic anhydride and a few drops of concentrated sulfuric acid were added and heated gently. Formation of a greenish or bluish coloration confirmed hydroxyl groups in triterpenoid acids [24].

#### Foam test (for saponins)

The resin infusion was dissolved in water and shaken vigorously. Persistent foam formation indicated the presence of saponins, which are sometimes present as minor constituents [25].

#### Fehling’s test (for reducing sugars, if present as impurities)

The resin infusion was dissolved in water and equal volumes of Fehling’s A and B solutions were added and heated gently. Formation of a red or orange precipitate indicated presence of reducing sugars [22].

#### Ferric chloride test (for phenolic compounds)

1-2 drops of 5% ferric chloride solution were added to both the resin solutions. A deep blue, green, or purple color indicated the presence of flavonoids [22].

#### Alkaloid tests

##### Dragendorff’s test

To the powdered drug or infusion, upon adding the Dragendorff’s reagent (Potassium iodide + Bismuth nitrate), appearance of orange red color precipitate indicated presence of alkaloids [22].

##### Mayer’s reagent test

To the powdered drug or infusion, upon adding the Mayer’s reagent (Potassium mercuric iodide) formation of cream color precipitate showed the presence of alkaloids [26].

##### Hager’s reagent test

To the powdered drug or infusion, upon adding the Hager’s reagent (saturated solution of picric acid), development of an orange yellow precipitate indicated presence of alkaloids [27].

##### Wagner’s reagent test

To the powdered drug or infusion, upon adding the Wagner’s (Iodine solution) reagent, formation of a brown flocculent precipitate indicated presence of alkaloids [28].

### Antioxidant activity

The antioxidant activities of frankincense infusions were determined using the 2,2-Diphenyl-1-picrylhydrazyl (DPPH) free radical scavenging assay. The samples were coded as Sample 1 (plain almond oil), Sample 2 (oil infusion of frankincense resin), Sample 3 (plain rosewater), and Sample 4 (rosewater infused frankincense resin). A 0.024 mg/mL solution of DPPH was prepared by dissolving 2.4 mg of DPPH in 100 mL methanol. Serial dilutions of each test sample (Sample 1 – Sample 4) and the standard antioxidant gallic acid were prepared in methanol to obtain different concentrations. For each assay, 5 µL of the respective sample or standard was added to 3.995 mL of the DPPH solution in a test tube. The reaction mixtures were mixed vigorously and incubated in the dark at room temperature for 30 min to allow complete reaction between the antioxidant compounds and the DPPH radicals. A control solution containing DPPH and methanol without sample or standard served as a blank to determine baseline absorbance. After incubation, the absorbance of each mixture was measured at 517 nm against blank using a UV-Vis spectrophotometer. All measurements were performed in triplicate to ensure accuracy and reproducibility and the results are expressed as mean ± standard deviation (SD) [29, 30]. Antioxidant activity was expressed as a percentage inhibition of DPPH radicals and calculated using the following equation:

$$\%\;Inhibition=\frac{A_{control\;}-A_{sample}}{A_{control}}\times 100$$

Where *A*_*control*_ is the absorbance of the DPPH solution without sample, and *A*_*sample*_ is the absorbance with the test sample.

### Formulation of *w/o* frankincense cream

A w/o emulsion-based cream was formulated by incorporating both oil and water infusions of frankincense. The formulation process included several stages starting from preparation of oil phase by combining oil infusion (30 g) with beeswax (7.5 g) and heating to 75 °C until the beeswax is melted completely. This was followed by preparation of aqueous phase where the water infusion (14–23 g, depending upon the formulation batch) was heated separately to the same temperature (Table 1). Eventually, the aqueous phase was added slowly into the oily phase with continuous stirring until both the phases were mixed properly. The cream formulation was removed from heat and kept stirring at room temperature until it was cooled and thickened, yielding a smooth and homogeneous *w/o* cream. A plain cream was also formulated following the same procedure using plain almond oil and rosewater, but without frankincense extracts.

**TABLE 1:** Ingredients and their proportions used in formulation development.

Ingredients	Weight by part	Weight in grams	Uses
Beeswax	1 part	7.5 g	Emulsifier
Sweet almond oil infusion	4 parts	30 g	Oily phase
Rosewater infusion	2/3 parts	14/23 g	Water phase

### Evaluation of cream formulations

The prepared cream formulations were subjected to a series of physicochemical evaluations [31].

#### Organoleptic properties

Physical properties such as appearance, color, odor, and texture of developed cream formulations were recorded visually.

#### Determination of pH

The pH was measured by dispersing 0.5 g of the cream formulation in 50 mL of distilled water and recording the value with a calibrated digital pH meter.

#### Viscosity

Viscosity of the developed cream formulation was determined using Brookfield viscometer (SHIMADZU, Japan) at 25 ± 0.5 °C following standard procedures reported for topical formulations [32], and the results were expressed in centipoise (cP). The rheological behaviour of the formulation was evaluated by recording viscosity at different spindle speeds.

#### Spreadability

Spreadability of the formulation was evaluated by the slip-and-drag method, measuring the time required for two glass slides with cream between them to slip under specified weight [33].

#### Phase separation

Cream formulations were stored at varying temperatures of 4 °C, 25 °C, and 40 °C and were inspected at set intervals (24 h, 7 days, 14 days, 28 days) for any signs of clear layers, oiling-out, or water pooling. Three freeze-thaw cycles were run and re-inspected.

#### Consistency

The consistency of the developed cream formulations was assessed by spreading the formulations on skin and observing its ease of application and feel.

#### Liquefaction

Cream samples were placed in marked vials at graded temperatures (40 – 60 °C) and inverted at defined times (0.5, 1, 2, 4, 24 h) and any continuous flow was observed. Time-to-flow and signs of oil pooling, if any, was recorded and the lowest temperature at which continuous flow begins, was noted.

#### Thermal behaviour

Heating-cooling stability of formulations was assessed at 4 °C for 24 h then 40 °C for 24 h per cycle for 3 consecutive cycles. Any visible changes or phase separation was observed after each cycle.

#### Grittiness

Grittiness of the formulation was assessed by gently rubbing the cream between fingers and observing under a microscope for the presence of coarse particles.

#### Washability

Washability was assessed by applying the cream formulation to skin and washing off with tap water, observing ease of removal.

#### Rheological behaviour

The rheological behaviour of cream formulation was measured with the help of Brookfield viscometer. 50 g of cream formulation was transferred to a 100 mL beaker and equilibrated at 25 ± 0.5 °C for 30 min. Suitable spindle was selected and immersed so that the cream covered the spindle mark. Viscosity values were recorded at different spindle speeds (5 – 100 rpm) to observe change in viscosity with shear rate [34].

#### Stability studies

The stability studies were carried out by storing the creams at different temperatures (4 °C, room temperature, and 40 °C) and under freeze-thaw cycles. Visual observations were made for phase separation, liquefaction, or other signs of instability.

#### FT-IR spectroscopy

The chemical characteristics of frankincense resin sample (S1), oil infusion (S2), water infusion (S3), plain cream formulation without frankincense (S4), and the cream formulation with combined frankincense infusions (S5) were evaluated using FT-IR spectroscopy to evaluate any possible interactions between the infusions and excipients. The FT-IR spectra were recorded over a transmittance range of 4,000 – 400 cm^−1^ and at a resolution of 4 cm^−1^ using silicone discs and major absorption peaks were assigned based on known characteristic frequencies.

## Results

### Phytochemical profiling

The qualitative phytochemical profiling of frankincense oil and water infusions was performed using standard qualitative tests for the detection of terpenoids, steroids, saponins, alkaloids, and phenolic compounds and the results revealed distinct differences in the types of secondary metabolites extracted into each medium (Table 2). The oil infusion exhibited strong positive reactions for triterpenoids and steroids as observed in the Salkowski test and Liebermann-Burchard test, which was consistent with the lipophilic nature of these compounds. The hydroxyl functional groups were also predominantly detected in the oil infusion by the acetylation test, reflecting the presence of alcohol-containing triterpenoids and boswellic acid derivatives. On the contrary, the water infusion showed a positive foam test indicating presence of saponins, as well as trace amounts of alkaloids as detected by the Dragendorff’s and Mayer’s reagent tests. Reducing sugars were also weakly detected, while phenolic compounds gave faint responses with the ferric chloride reagent. These findings suggested that the oil infusion consisted primarily of the lipophilic terpenic and resinous components, while the water infusion showed presence of hydrophilic polysaccharides, saponins, and small amounts of alkaloids.

**TABLE 2:** Results of phytochemical analysis of oil and water infusions of frankincense resin.

S. No	Phytochemicals	Test	Blank 1 (rosewater only)	Water infusion of resin	Blank 2 (almond oil only)	Oil infusion of resin
1	Triterpenoids and steroids	Salkowski test	−	−	+++	+++
2	Triterpenoids	Liebermann-Burchard test	−	−	−	+++
3	Hydroxyl groups in boswellic acids	Acetylation test	−	−	+	+
4	Saponins	Foam test	−	+++	+	+
6	Reducing sugars	Fehling’s test	−	+	−	−
6	Carbohydrates	Molisch test	−	+	−	+
		Resorcinol test	−	+	−	+
7	Phenolic compounds	Ferric chloride test	−	+	−	−
8	Alkaloids	Dragendorff’s test	−	+	−	−
		Mayer’s test	−	+	−	−
		Hager’s test	−	+	−	−
		Wagner’s test	−	+	−	−

### Antioxidant activity

**FIGURE 2:**
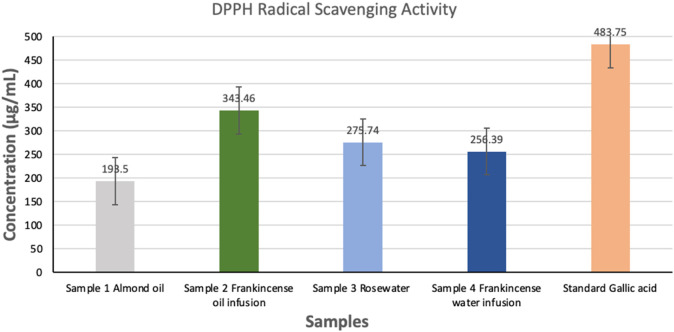
DPPH radical scavenging activity of different samples 1-4 and standard gallic acid (positive control). Values are expressed as mean ± SD (n = 3). Sample 1: plain almond oil; sample 2: almond oil infusion of frankincense resin; sample 3: plain rosewater; and sample 4: rosewater infusion of frankincense resin.

The DPPH assay is a widely used method to evaluate the free radical scavenging ability of compounds. The antioxidant potential of four treatment groups was evaluated based on their concentration (µg/mL) using the DPPH radical scavenging assay and the results are shown in Figure 2. The percentage inhibition of DPPH radicals and the corresponding antioxidant concentrations (expressed in µg/mL) for four different samples, labeled as sample 1 (plain almond oil), sample 2 (oil infusion of frankincense resin), sample 3 (plain rosewater), and sample 4 (rosewater infusion of frankincense resin) are presented in Table 3. Among the samples, sample 2 exhibited highest antioxidant activity with a 71% inhibition and a corresponding concentration of 343.46 ± 34.2 μg/mL, indicating strong free radical scavenging ability of the sample. Sample 4 and sample 3 also showed good antioxidant activities with inhibition rates of 57% and 53%, and concentrations of 275.74 ± 46.4 μg/mL and 256.39 ± 38.9 μg/mL, respectively. Sample 1 showed the lowest antioxidant activity, with a 40% inhibition and a concentration of 193.50 ± 28.1 μg/mL. As evident from the results, a positive correlation between % inhibition and the antioxidant concentration were observed, suggesting that higher concentrations were associated with greater free radical scavenging activity. Results showed varying degrees of antioxidant potential among the samples, with sample 2 demonstrating the most potent activity. Overall antioxidant activity of tested samples followed the order: sample 2 > sample 4 > sample 3 > sample 1. These differences highlighted the potential influence of different phytochemical compositions of the samples. Strikingly high activity in sample 2 makes it a promising candidate for applications related to oxidative stress or as a natural antioxidant source in pharmaceutical or nutraceutical formulations.

**TABLE 3:** Percentage inhibition and corresponding antioxidant concentrations of tested samples as determined by the DPPH assay.

Samples*	% inhibition	Concentration (µg/mL) Mean ± SD
Sample 1	40	193.50 ± 28.1
Sample 2	71	343.46 ± 34.2
Sample 3	53	256.39 ± 38.9
Sample 4	57	275.74 ± 46.4
Standard (gallic acid)	97	483.75 ± 27.4

* Sample 1: plain almond oil; sample 2: almond oil infusion of frankincense resin; sample 3: plain rosewater; and sample 4: rosewater infusion of frankincense resin.

### Evaluation of cream formulation

The plain and combined infusion cream formulations were analyzed on the basis of various evaluation parameters to ensure the development of formulation with desired properties. The observations are summarized in Table 4.

**TABLE 4:** Physicochemical evaluation of plain and combined infusion cream formulations.

Parameters	Plain formulation	Combined infusion formulation
Color	White	Fawn
Feel	Smooth	Smooth
Odour	Odourless	Pleasant
Homogeneity	Satisfying	Homogenous
pH	7.55	7.28
Viscosity (cP)	1350	1200
Phase separation	Little phase separation	No phase separation
Consistency	Good	Softer
Liquefaction	Immediate	Delayed
Thermal behaviour	Found in stable state at room temperature (25 °C), refrigerator (4 °C) and at 40 °C for 3 consecutive cycles	Found in stable state at room temperature (25 °C), refrigerator (4 °C) and at 40 °C for 3 consecutive cycles
Grittiness	None	None
Spreadability	8–10 s	7 s
Washability	Difficult to wash	Easily washable

#### Organoleptic properties

The plain formulation exhibited a stark white color, while the infusion-based formulation showed a more appealing fawn color. This color variation is attributed to the presence of natural pigments in the infusions, which could impart a subtle color to the final product. Both the formulations showed smooth texture, indicating desirable tactile experience for the end-user. A smooth texture is crucial for cosmetic products, as it ensures easy application and minimizes discomfort during use. A significant difference was observed in the odor profile of both the products as the plain formulation was odourless, while the infusion-based formulation had a pleasant aroma owing to the presence of volatile oils and terpenoids in the infusion. Both formulations demonstrated satisfactory level of homogeneity, suggesting uniform distribution of ingredients throughout the product. A homogeneous product ensured consistent performance and prevented the separation of components, which is crucial for maintaining product stability and efficacy.

#### Determination of pH

The plain cream formulation exhibited a pH of 7.55, while the infusion-based formulation displayed a slightly lower pH of 7.28, which falls within the acceptable range for topical formulations, though slightly higher than the natural skin surface pH.

#### Viscosity

The viscosity of frankincense cream was observed to be lesser than the plain cream formulation, and was calculated to be approximately 1,200 cP, which indicated adequate thickness and stability suitable for topical applications. The rheological behaviour observed was shear-thinning, which is typical for non-Newtonian fluids and crucial for the ease of application. It is desirable for cream formulations as it allows easy spreading under mechanical stress while maintaining stability at rest.

#### Spreadability

Spreadability was tested by spreading the formulations between glass slides under applied weight and it was observed that the creams had variable spreading patterns. Plain formulation (8–10 s) took longer to spread than the infusion-based formulation (7 s).

#### Phase separation

**FIGURE 3:**
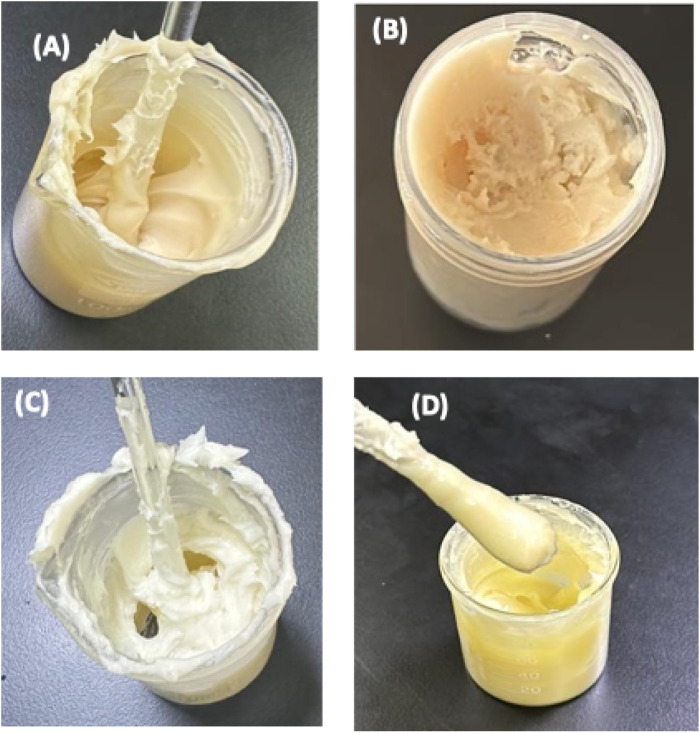
Photographic representation of stability evaluation of the frankincense cream, formulation under different storage conditions including **(A)** Day 0 at room temperature; **(B)** under refrigerated conditions (4 °C); **(C)** plain cream formulation at day 0 at room temperature; and **(D)** frankincense cream formulation at accelerated temperature 40 °C.

In contrast to the plain cream formulation where a little phase separation was observed, the combined infusion-based formulation demonstrated no visible phase separation during the stability tests, suggesting good compatibility of the oil and water phases with the emulsifiers used (Figure 3). Incorporating natural resins and plant extracts stabilized the cream matrix, which might be due to the interaction between emulsifiers and active components [35].

#### Consistency

The addition of frankincense infusion resulted in a slightly softer consistency compared to the plain formulation, which might be due to the resin’s unique composition of polysaccharides and volatile oils. This was also influenced by the lipid content, emulsifiers, and the effect of infusions on the cream matrix.

#### Liquefaction

The extract formulation showed delayed liquefaction compared to the plain formulation, indicating improved thermal stability. Studies involving other herbal-based creams, such as those formulated with *Lavandula angustifolia* and *Aloe vera* also reported enhanced stability due to the presence of bioactive compounds that reinforce the structural matrix [36, 37].

#### Thermal behaviour

The incorporation of frankincense infusion to the cream formulation improved the its thermal behaviour, as evidenced by its stability over a wide range of temperatures. The interaction between the resin’s boswellic acids and the cream’s lipid phase might have contributed to the enhanced thermal resilience (Figure 3D). It was consistent with the findings reported for the formulations containing natural resins and gums, where the complex molecular structures provided thermal resistance [38].

#### Grittiness

No gritty particles were found in both the formulations while observing under the microscope and when pressed between fingers.

#### Washability

The frankincense infusion-based cream could easily be removed upon washing, while the plain formulation was comparatively difficult to wash.

#### Rheological behaviour

**FIGURE 4:**
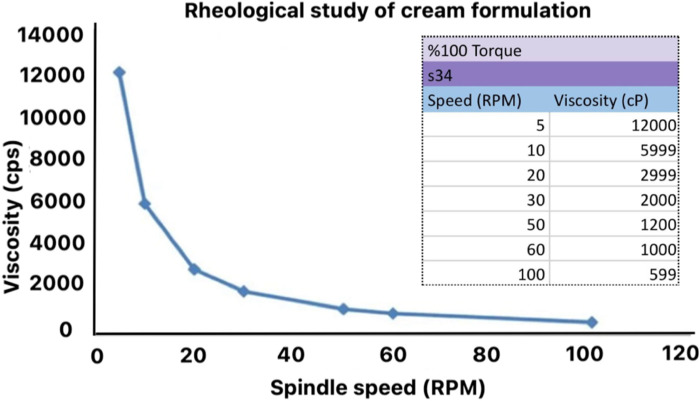
Rheological behaviour of frankincense cream formulation showing effect of spindle speed on viscosity using Brookfield viscometer at 25 °C. The decrease in viscosity with increasing spindle speed indicated pseudoplastic (shear-thinning) behaviour of the formulation.

Creams are characterized by their semi-solid nature and respond differently when subjected to stress (force). The cream sample under examination was identified as shear-thinning, as a decrease in viscosity was observed with an increase in stress. This behaviour was a prevalent form of time-independent non-Newtonian fluid dynamics, which is closely associated with the spreadability of cosmetics when applied to human skin [39] (Figure 4).

#### FT-IR spectroscopic analysis

**FIGURE 5:**
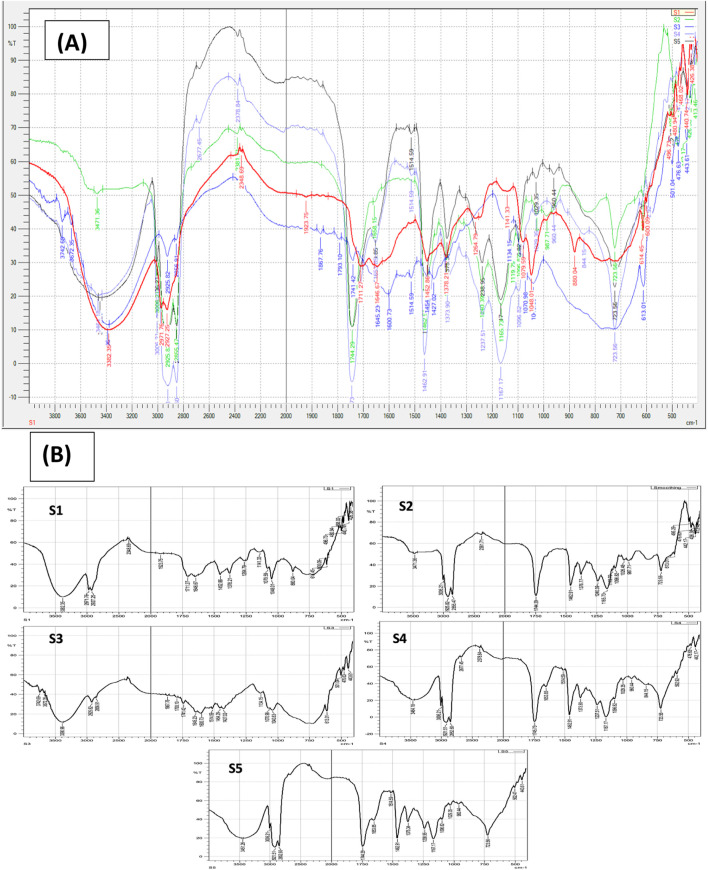
**(A)** Overlay; and **(B)** stacked FT-IR spectra of pure frankincense resin (S1), oil infusion (S2), water infusion (S3), plain cream formulation (S4) and combined infusion formulation (S5). The preservation of characteristic absorption peaks indicated successful incorporation of frankincense constituents into the cream matrix without chemical interaction.

The overlay and stacked FT-IR spectra of samples in the order S1, S2, S3, S4, S5 at mid infrared (IR) region (4,000-500 cm^−1^) are shown in Figures 5A,B. In this study, the selection of samples relied on its availability in the cream formulation. The spectra of S4 (plain cream formulation without resin) and S5 (final cream formulation with resin) appeared to be similar indicating no interaction between the natural components and appearance of typical characteristic of absorption bands. Each peak in FT-IR spectra corresponded to functional groups responsible for IR absorption. In S1 (pure frankincense resin), FT-IR spectrum exhibited an absorption peak at 3,382 cm^-1^ caused by asymmetric stretching vibrations of -OH functional group. Several other bands were also observed at 2,971, 1711, 1,452, 1,378, 1,264, 1,048 and 880 cm^−1^ which can be attributed to stretching vibrations -C-H, -C=O stretching vibrations, N-H bending vibrations in a plane, C-H bending vibrations, C-O stretching vibrations, C-C bending vibrations, and N-H out-of-plane bending vibrations respectively. These results were concordant with the FT-IR analysis of frankincense resin present in the literature [40]. In S2 (almond oil infusion of frankincense resin), absorption peaks were observed at 3,471, 2,925, 1744, 1,462, 1,376, 1,240, 1,026 and 724 cm^−1^. This clearly indicated that the oil soluble components from resin were successfully infused in plain almond oil. In S3 (Madinah rosewater infusion of frankincense resin), modified absorption peaks were observed at 3,390, 2,925, 1867, 1741, 1,645, 1,514, 1,454, 1,427, 1,070 cm^−1^. These could be due to the presence of water-soluble resin components in rosewater.

Sample S4 (plain cream formulation without frankincense resin) showed no absorption band characteristic to frankincense resin indicating its absence from the formulation. Lastly, in S5 (frankincense cream formulation containing combined infusions of resin), absorption bands were found intact at 3,451, 2,921, 1744, 1,462, 1,375, 1,238, 1,029, and 723 cm^−1^ indicating the presence of all functional groups corresponding to the frankincense resin. It also showed peaks at 1,653, 1,514 cm^-1^ similar to the S3 sample. The purpose of this study was to ensure that the final frankincense resin-based cream formulation possessed all the oil and water-soluble resinous components of frankincense for maximum skincare activity and no interaction was observed between the frankincense constituents and the cream base. The cream matrix maintained the structural integrity of the active phytochemicals. These findings support the successful incorporation of both hydrophilic and lipophilic components of frankincense into the *w/o* cream formulation without compromising their functional groups.

## Discussion

The present study focuses on identification, qualitative chemical profiling and a conventional cream formulation development of combined infusions of frankincense resin. It sought to combine the traditional knowledge of frankincense use with modern phytochemical and formulation approaches, resulting in the successful development of *w/o* nourishing and anti-aging cream containing both oil- and water-soluble fractions of the resin. The two infusions showed distinct phytochemical compositions, contributing to antioxidant activity and stability of the developed formulation, thereby providing a strong scientific rationale for its potential application as a dermocosmetic agent. An all-natural skincare formulation was developed which consisted of pure frankincense resin infusion, pure almond oil, beeswax and Madinah rosewater as main ingredients. Frankincense essential oil has been reported to have anti-inflammatory, antibacterial and antioxidant properties that benefit all skin types, including acne and blemish-prone skin, wrinkles, and dry skin [15].

Essential oils and plant-derived extracts have garnered increasing attention in pharmaceutical and cosmeceutical research owing to their diverse biological effects including antioxidant, antimicrobial, and anti-inflammatory. These natural products have long been widely investigated for their potential applications in formulations intended for dermatological and skin-care products [41, 42]. Frankincense essential oil is known to stimulate new cell production, preserves skin elasticity, and relieves dry and chapped skin [43]. It is an effective antiwrinkle and antiaging agent that may also be used to treat psoriasis and dermatitis [44, 45]. Beeswax is a hydrophobic compound, which is structurally a complex mixture of alkanes, alkenes, free fatty acids, monoesters, diesters, and hydroxy-monoesters, and is useful in skin care products as natural a natural emulsifier [46]. It is generally an essential part of any cream formulation as it thickens the cream and keeps the oil and water phases of the cream from separating. It also acts as an occlusive, which helps to create a semi-occlusive skin barrier that minimizes trans-epidermal water loss; an emollient, which softens and soothes the skin; and a humectant, which locks in hydration in the skin [47]. High amounts of propolis in beeswax contribute to its antimicrobial [48], antioxidant [49], and anti-inflammatory properties [50]. Almond oil is widely used to soothe and moisturize the skin. It is also rich in vitamin E, which helps protect the skin from harmful UV radiation. Therapeutic properties of almond oil include anti-inflammatory, immune-stimulating, and anti-hepatotoxic activities [51]. Madinah rosewater was used instead of plain water as it has a cooling impact on the skin, has good aroma, and rose extracts themselves are considered as a good source of antioxidants. Additionally, it has antibacterial effects on a variety of microflora, anti-inflammatory, moisturizing, and soothing properties [52].

Qualitative chemical analysis confirmed that the oil infusion was rich in triterpenoids and related boswellic acid-type constituents, whereas the water infusion consisted primarily of saponins, polysachharides, and trace amount of alkaloids. The detection of hydroxyl and carbonyl functional groups in FT-IR spectra further supported the presence of boswellic acids and associated terpenoids in the oil extract. Incorporating both the fractions in a single formulation may allow for broader therapeutic coverage as the anti-inflammatory and antioxidante effects of triterpenoids are combined with the moisturizing and emulsifying properties of polysaccharides and saponins. Phytochemicals from *B. serrata* and *B. carterii* were identified previously, where the ratio of AKβ-BA (acetyl-11-keto-β- boswellic acid) to Kβ-BA (11-keto-β-boswellic acid) differed significantly [53]. In another similar study, it was revealed that the resinous portion of *B. serrata* contained monoterpenes, diterpenes, triterpenes, tetracyclic triterpenic acids, and four main pentacyclic triterpenic acids, which were responsible for inhibiting pro-inflammatory enzymes [54]. Acetyl-11-keto-β-boswellic acid was the most effective of these boswellic acids at inhibiting the inflammation-causing enzyme 5-lipoxygenase.

The DPPH radical scavenging assay demonstrated that the almond oil infusion of frankincense resin possessed the highest antioxidant activity, followed by the rosewater infusion, plain rosewater, and plain almond oil. The stronger antioxidant action of the oil infusion could be attributed to the abundance of lipophilic boswellic acids and terpenoids in the infusion, many of which have been reported to neutralize free radicals and modulate oxidative pathways. While the rosewater infusion exhibited comparatively weaker antioxidant activity, its inclusion in the formulation will further contribute to its overall antioxidant potential. Any skincare formulation containing bioactives of high antioxidant potential help neutralise the free radicals, thereby preventing the damage caused by reactive oxygen and reactive nitrogen species. The antioxidant potential of frankincense resin is well documented in the literature [55, 56].

Anti-photoaging effect of frankincense (*B*. *papyrifera* (Del.) Hochst., family Burceraceae) was studied recently in an *in vivo* model. Superior anti-aging effect of frankincense oil were observed compared to epigallocatechin gallate. The frankincense oil was subsequently formulated in solid lipid nanoparticles through high shear homogenization process to improve solubility and skin penetration characteristics [15]. In another study, antioxidant activity of frankincense oil was evaluated with DPPH and ABTS methods with the resulting inhibition of 73.88 ± 0.35% of DPPH radical and 97.09 ± 0.48% of ABTS radical cation was reported [57]. In another study reported earlier, antioxidant properties of various solvent extracts (methanol, ethanol, acetone, water) of Indian frankincense (*B. serrata*) oleogum resin were carried out by DPPH radical scavenging activity, reducing power assay, total antioxidant capacity, and oxidative stability index (Rancimat test). Various solvent extracts of *B. serrata* showed varying degrees of antioxidant activity in different test systems in a concentration dependent manner. Methanol was found to be the most efficient solvent for extraction of antioxidants from *B. serrata* and exhibited strongest antioxidant potential in all assays used [58].

Apart from having a high antioxidant potential, frankincense resin also has anti-inflammatory, antifungal and antibacterial properties, offering new possibilities for its use as an active skincare agent. TNFα, IL-1β, and IL-6 are examples of pro-inflammatory cytokines that are crucial to the inflammatory response and the regulation of both acute and chronic inflammation is significantly influenced by these inflammatory cytokines [59]. Monoterpenes, diterpenes, triterpenes, tetracyclic triterpenic acids, and four primary pentacyclic triterpenic acids—l-boswellic acid, acetyl-l-boswellic acid, 11-keto-l-boswellic acid, and acetyl-11-keto-l-boswellic acid—are all present in the resinous portion of *B. serrata*, which are known to downregulate these pro-inflammatory cytokines [54].

The *w/o* cream formulation prepared using combined oil- and water-infusions of frankincense resin demonstrated favourable physicochemical properties suitable for topical use, including smooth texture, uniformity, viscosity and an acceptable pH of 7.28. Notably, stability studies revealed no evidence of phase separation, liquefaction, or grittiness under different thermal conditions and freeze-thaw cycles, indicating the robustness of the emulsion system. Rheological assessment indicated shear-thinning behaviour, which is a desirable attribute of a cream formulation ensuring ease of spread upon application while maintaining stability at storage. The FT-IR analysis of infusions and formulations provided important data about the integrity of chemical composition as major functional groups of frankincense resin were retained in the final cream formulation. The active constituents remained intact within the cream base and no significant interactions with excipients were noticed. Comprehensive biological evaluation of plant-derived bioactive components often requires a combination of *in vitro*, *ex vivo*, and *in vivo* studies to fully establish their therapeutic potential [60].

### Limitations of the study and future directions

Despite promising results, this study has several limitations as it represents a preliminary formulation development and *in vitro* evaluation study. The phytochemical characterization of the infusions was limited to qualitative screening; therefore, future investigations are warranted to quantify specific boswellic acids, terpenes, and polysaccharides using advanced chromatographic methods such as HPLC, LC-MS, or GC-MS, which would further help correlate phytochemical constituents with the observed antioxidant activity. Similarly, the antioxidant potential was assessed only by the DPPH assay, which, although widely employed, represents a chemical model rather than having biological relevance. Complementary assays and *in vitro* cell-based antioxidant or anti-inflammatory models could be performed. Future studies should also include cytotoxicity assays using keratinocyte or fibroblast cell lines together with the skin compatibility studies to further examine the dermatological applicability of the developed formulation. Molecular docking analysis, cell-based assays, and mechanistic investigations can also be performed to better understand the interaction of boswellic acids and related phytoconstituents with molecular targets involved in the oxidative stress and skin aging pathways. Statistical optimization approaches such as factorial design and response surface methodology can be applied for the development and optimization of formulation to improve drug delivery performance. Additionally, a pH of 7.28 is although in a tolerable range of pH of topical formulation, it is slightly higher than the skin surface pH (∼5.5) and this could have effect on long-term tolerability. Further formulation adjustments are needed to bring the cream closer to the skin pH. Lastly, formal *in vivo* and *ex vivo* evaluation such as skin permeation studies, and irritancy/sensitization testing are essential to substantiate the claims of anti-aging and dermocosmetic efficacy. Future investigation may also explore advanced delivery systems and permeation-enhancing formulations to further improve dermal bioavailability of frankincense constituents. Considering the reported antimicrobial properties of frankincense resin, future investigations may also focus on assessing antibacterial activity of the developed formulation against common skin pathogens. These additional investigations are needed to be performed to establish the pharmacological relevance, safety profile, and dermal bioavailability of the bioactive components of frankincense prior to clinical applications.

### Conclusion

In conclusion, the present study demonstrated extraction, characterization, and development of a stable *w/o* cream formulation incorporating a combined extract of frankincense resin, holding great promise for potential dermatological applications. The formulation has successfully eliminated commonly used ingredients such as alcohol, mineral oil, sodium lauryl sulfate, parabens, phthalates, and propylene glycol, making it a desirable option for individuals seeking all-natural skincare products free from these substances. The developed formulation showed favorable physicochemical characteristics and encouraging antioxidant activity, indicating its potential application as natural dermocosmetic preparation for skin protection against oxidative stress and photo-aging. However, further dermatological studies are warranted to evaluate the formulation’s safety on the skin, specifically to assess its potential for causing skin irritation and erythema. While encouraging, the present work represents a preliminary step in the scientific validation of frankincense-based formulations. With its dual oil- and water-soluble bioactive fractions, frankincense resin provides a unique opportunity in the development of multifunctional, natural anti-aging creams. Future comprehensive studies shall provide valuable insights into the formulation’s suitability for use in skincare products and ensure its compatibility with the skin, reinforcing its potential as a safe and effective ingredient.

## Data Availability

The original contributions presented in the study are included in the article/supplementary material, further inquiries can be directed to the corresponding author.

## References

[1] Abdel-Tawab M Werz O Schubert-Zsilavecz M . *Boswellia serrata*: an overall assessment of *in vitro,* preclinical, pharmacokinetic and clinical data. Clin Pharmacokinet (2011) 50(6):349–69. 10.2165/11586800-000000000-00000 21553931

[2] Al-Saidi S Rameshkumar KB Hisham A Sivakumar N Al-Kindy S . Composition and antibacterial activity of the essential oils of four commercial grades of Omani luban, the oleo-gum resin of Boswellia sacra FLUECK. Chem Biodivers (2012) 9(3):615–24. 10.1002/cbdv.201100189 22422529

[3] Marongiu B Piras A Porcedda S Tuveri E . Extraction of *Santalum album* and *Boswellia carterii* Birdw. volatile oil by supercritical carbon dioxide: influence of some process parameters. Flavour Frag J (2006) 4:718–24. 10.1002/ffj.1718

[4] Al-Yasiry AR Kiczorowska B . Frankincense--therapeutic properties. Postepy Hig Med Dosw (Online) (2016) 70:380–91. 10.5604/17322693.1200553 27117114

[5] Mohamed AA Ali SI EL-Baz FK Hegazy AK Kord MA . Chemical composition of essential oil and *in vitro* antioxidant and antimicrobial activities of crude extracts of *Commiphora myrrha* resin. Ind Crop Prod (2014) 57:10–6. 10.1016/j.indcrop.2014.03.017

[6] Miran M Amirshahrokhi K Ajanii Y Zadali R Rutter MW Enayati A Taxonomical investigation, chemical composition, traditional use in medicine, and pharmacological activities of *Boswellia sacra* flueck. Evid Based Complement Alternat Med (2022) 2022:8779676. 10.1155/2022/8779676 35222678 PMC8881160

[7] Assefa M Dekebo H Kassa H Habtu A Fitwi G Redi-Abshiro M . Biophysical and chemical investigations of frankincense of *Boswellia papyrifera* from north and northwestern Ethiopia. J Chem Pharm Res (2012) 4:1074–89.

[8] Baser KHC Demirci B Dekebo A Dagne E . Essential oils of some *Boswellia* spp., Myrrh and Opopanax. Flavour Frag J (2003) 18(2):153–6. 10.1002/ffj.1166

[9] Ragab EA Abd El-Wahab MF Doghish AS Salama RM Eissa N Darwish SF . The journey of boswellic acids from synthesis to pharmacological activities. Naunyn Schmiedebergs Arch Pharmacol (2024) 397(3):1477–504. 10.1007/s00210-023-02725-w 37740772 PMC10858840

[10] Ahmed M Ali D Harrath AH Hussain T Al-Daghri N Alokail MS Ultrastructural and hormonal changes in rat cauda epididymal spermatozoa induced by *Boswellia papyrifera* and *Boswellia carterii* . C R Biol (2014) 337(4):250–7. 10.1016/j.crvi.2014.01.007 24702894

[11] Xia D Lou W Fung KM Wolley CL Suhail MM Lin HK . Cancer chemopreventive effects of Boswellia sacra gum resin hydrodistillates on invasive urothelial cell carcinoma: report of a case. Integr Cancer Ther (2017) 16(4):605–11. 10.1177/1534735416664174 27531547 PMC5739138

[12] Khan F Rashan L . Phytochemical analysis and pharmaceutical applications of monoterpenoids present in the essential oil of *Boswellia sacra* (Omani Luban). Adv Pharmacol Pharm Sci (2025) 2025:3536898. 10.1155/adpp/3536898 40040632 PMC11876528

[13] Banno N Akihisa T Yasukawa K Tokuda H Tabata K Nakamura Y Anti-inflammatory activities of the triterpene acids from the resin of Boswellia carteri. J Ethnopharmacol (2006) 107(2):249–53. 10.1016/j.jep.2006.03.006 16621377

[14] Roy NK Parama D Banik K Bordoloi D Devi AK Thakur KK An update on pharmacological potential of boswellic acids against chronic diseases. Int J Mol Sci (2019) 20(17):4101. 10.3390/ijms20174101 31443458 PMC6747466

[15] Kotb EA El-Shiekh RA Abd-Elsalam WH El Sayed NSED El Tanbouly N El Senousy AS . Protective potential of frankincense essential oil and its loaded solid lipid nanoparticles against UVB-induced photodamage in rats *via* MAPK and PI3K/AKT signaling pathways; A promising anti-aging therapy. PLoS One (2023) 18(12):e0294067. 10.1371/journal.pone.0294067 38127865 PMC10735031

[16] Han X Rodriguez D Parker TL . Biological activities of frankincense essential oil in human dermal fibroblasts. Biochim Open (2017) 4:31–5. 10.1016/j.biopen.2017.01.003 29450138 PMC5801908

[17] Togni S Maramaldi G Di Pierro F Biondi M . A cosmeceutical formulation based on boswellic acids for the treatment of erythematous eczema and psoriasis. Clin Cosmet Investig Dermatol (2014) 7:321–7. 10.2147/CCID.S69240 25419153 PMC4235203

[18] Wang MX Zhao JX Meng YJ Di TT Xu XL Xie XJ Acetyl-11-keto-β-boswellic acid inhibits the secretion of cytokines by dendritic cells via the TLR7/8 pathway in an imiquimod-induced psoriasis mouse model and *in vitro* . Life Sci (2018) 207:90–104. 10.1016/j.lfs.2018.05.044 29859222

[19] Shamraiz U Hussain H Ur Rehman N Al-Shidhani S Saeed A Khan HY Synthesis of new boswellic acid derivatives as potential antiproliferative agents. Nat Prod Res (2020) 34(13):1845–52. 10.1080/14786419.2018.1564295 30691289

[20] Verma M Fatima S Saeed M Ansari IA . Anti-proliferative, pro-apoptotic, and chemosensitizing potential of 3-acetyl-11-keto-β-boswellic Acid (AKBA) against prostate cancer cells. Mol Biotechnol (2025) 67(2):746–61. 10.1007/s12033-024-01089-7 38502429

[21] Kang SY Um JY Chung BY Lee SY Park JS Kim JC Moisturizer in patients with inflammatory skin diseases. Medicina (Kaunas) (2022) 58(7):888. 10.3390/medicina58070888 35888607 PMC9315586

[22] Das BK Al-Amin MM Russel SM Kabir S Bhattacherjee R Hannan JM . Phytochemical screening and evaluation of analgesic activity of Oroxylum indicum. Indian J Pharm Sci (2014) 76(6):571–5. 25593396 PMC4293694

[23] Rahimah SB Djunaedi DD Soeroto AY Bisri T . The phytochemical screening, total phenolic contents and antioxidant activities *in vitro* of white oyster mushroom (Pleurotus ostreatus) preparations. Open Access Maced J Med Sci (2019) 7(15):2404–12. 10.3889/oamjms.2019.741 31666837 PMC6814466

[24] Mannino G Occhipinti A Maffei ME . Quantitative determination of 3-O-Acetyl-11-Keto-βBoswellic acid (AKBA) and other boswellic acids in Boswellia sacra flueck (syn. B. carteri birdw) and Boswellia serrata roxb. Molecules (2016) 21(10):1329. 10.3390/molecules21101329 27782055 PMC6273064

[25] Chen YF Yang CH Chang MS Ciou YP Huang YC . Foam properties and detergent abilities of the saponins from Camellia oleifera. Int J Mol Sci (2010) 11(11):4417–25. 10.3390/ijms11114417 21151446 PMC3000090

[26] Kancherla N Dhakshinamoothi A Chitra K Komaram RB . Preliminary analysis of phytoconstituents and evaluation of anthelminthic property of Cayratia auriculata *(in vitro)* . Maedica (Bucur) (2019) 14(4):350–6. 10.26574/maedica.2019.14.4.350 32153665 PMC7035446

[27] Godlewska K Pacyga P Szumny A Szymczycha-Madeja A Wełna M Michalak I . Methods for rapid screening of biologically active compounds present in plant-based extracts. Molecules (2022) 27(20):7094. 10.3390/molecules27207094 36296683 PMC9612180

[28] Dahanayake JM Perera PK Galappatty P Perera HDSM Arawwawala LDAM . Comparative phytochemical analysis and antioxidant activities of Tamalakyadi decoction with its modified dosage forms. Evid Based Complement Alternat Med (2019) 2019:6037137. 10.1155/2019/6037137 31186663 PMC6521515

[29] Menachery SJ Moni SS Khardali A Elmobark ME Alam MF Ali Albarqi AA Exploring the bioactive constituents, antibacterial, antioxidant potentialities of *Aloe officinalis* Forssk flower. Nat Prod Commun (2025) 20(1):1934578X251314723. 10.1177/1934578X251314723

[30] Baliyan S Mukherjee R Priyadarshini A Vibhuti A Gupta A Pandey RP Determination of antioxidants by DPPH radical scavenging activity and quantitative phytochemical analysis of *Ficus religiosa* . Molecules (2022) 27(4):1326. 10.3390/molecules27041326 35209118 PMC8878429

[31] Bardaa S Makni K Boudaouara O Bardaa T Ktari N Hachicha S Development and evaluation of the wound healing effect of a novel topical cream formula based on *Ginkgo biloba* extract on wounds in diabetic rats. Biomed Res Int (2021) 2021:6474706. 10.1155/2021/6474706 34692837 PMC8528584

[32] Khan BA Ahmad S Khan MK Hosny KM Bukhary DM Iqbal H Fabrication and characterizations of pharmaceutical emulgel Co-Loaded with naproxen-eugenol for improved analgesic and anti-inflammatory effects. Gels (2022) 8(10):608. 10.3390/gels8100608 36286109 PMC9602183

[33] Sharif A Akhtar N Khan MS Menaa A Menaa B Khan BA Formulation and evaluation on human skin of a water-in-oil emulsion containing Muscat hamburg black grape seed extract. Int J Cosmet Sci (2015) 37(2):253–8. 10.1111/ics.12184.16 25402429

[34] Akhtar N Menaa F Akhtar N Javed N Sethi A Khan MS . Tocopherol succinate-loaded ethosomal gel synthesized by cold method technique: deeper biophysical characterizations for translational application on human skin. J Cosmet Dermatol (2024) 23(3):1015–28. 10.1111/jocd.16054 38268219

[35] Michalak M . Plant extracts as skin care and therapeutic agents. Int J Mol Sci (2023) 24(20):15444. 10.3390/ijms242015444 37895122 PMC10607442

[36] Joshi P Joshi S Rajani U Semwal RB Semwal DK . Formulation and evaluation of polyherbal cream and lotion for the treatment of psoriasis-induced secondary infections. Curr Rev Clin Exp Pharmacol (2021) 16(1):79–96. 10.2174/1574884714666191017111218 31622222

[37] Ahshawat MS Saraf S Saraf S . Preparation and characterization of herbal creams for improvement of skin viscoelastic properties. Int J Cosmet Sci (2008) 30(3):183–93. 10.1111/j.1468-2494.2008.00442.x 18452435

[38] Amiri MS Mohammadzadeh V Yazdi MET Barani M Rahdar A Kyzas GZ . Plant-based gums and mucilages applications in pharmacology and nanomedicine: a review. Molecules (2021) 26(6):1770. 10.3390/molecules26061770 33809917 PMC8004199

[39] Moldovan M Lahmar A Bogdan C Părăuan S Tomuţă I Crişan M . Formulation and evaluation of a water-in-oil cream containing herbal active ingredients and ferulic acid. Clujul Med (2017) 90(2):212–9. 10.15386/cjmed-668 28559707 PMC5433575

[40] Mohamed N Ismail H Nasr GM Abdel-Ghany S Arneth B Sabit H . Anti-tumor potential of frankincense essential oil and its nano-formulation in breast cancer: an *in vivo* and *in vitro* study. Pharmaceutics (2025) 17(4):426. 10.3390/pharmaceutics17040426 40284420 PMC12030047

[41] Meziane H Zraibi L Albusayr R Bitari A Oussaid A Hammouti B Rosmarinus officinalis Linn.: unveiling its multifaceted nature in nutrition, diverse applications, and advanced extraction methods. J Umm Al-qura Univ Appl Sci (2025) 11:9–37. 10.1007/s43994-024-00144-y

[42] Diass K Oualdi I Benabbas R Zaki H Ouabane M Hammouti B Use of essential oils for the treatment of Fusarium oxysporum f. sp. Albedinis: Chemical profile, *in vitro* antifungal activity, and *in silico* investigation by molecular docking study. Curr Chem Biol (2024) 18(4):193–214. 10.2174/0122127968296919240926095348

[43] Venkatesan K Sivadasan D Abderrahmen AWM Gayasuddin Mouid M Goyal M Bansal M Protective effects of frankincense oil on wound healing: downregulating caspase-3 expression to facilitate the transition from the inflammatory to proliferative phase. Pharmaceuticals (Basel) (2025) 18(3):407. 10.3390/ph18030407 40143183 PMC11945088

[44] Halim SA Khan A Csuk R Al-Rawahi A Al-Harrasi A . Diterpenoids and triterpenoids from frankincense are excellent anti-psoriatic agents: an *in silico* approach. Front Chem (2020) 8:486. 10.3389/fchem.2020.00486 32671018 PMC7330179

[45] Javed S Mangla B Salawi A Sultan MH Almoshari Y Ahsan W . Essential oils as dermocosmetic agents, their mechanism of action and nanolipidic formulations for maximized skincare. Cosmetics (2024) 11(6):210. 10.3390/cosmetics11060210

[46] Nong Y Maloh J Natarelli N Gunt HB Tristani E Sivamani RK . A review of the use of beeswax in skincare. J Cosmet Dermatol (2023) 22(8):2166–73. 10.1111/jocd.15718 36999457

[47] Pavlačková J Egner P Slavík R Mokrejš P Gál R . Hydration and barrier potential of cosmetic matrices with bee products. Molecules (2020) 25(11):2510. 10.3390/molecules25112510 32481539 PMC7321148

[48] Przybyłek I Karpiński TM . Antibacterial properties of propolis. Molecules (2019) 24(11):2047. 10.3390/molecules24112047 31146392 PMC6600457

[49] Kurek-Górecka A Rzepecka-Stojko A Górecki M Stojko J Sosada M Swierczek-Zieba G . Structure and antioxidant activity of polyphenols derived from propolis. Molecules (2013) 19(1):78–101. 10.3390/molecules19010078 24362627 PMC6271064

[50] Wagh VD . Propolis: a wonder bees product and its pharmacological potentials. Adv Pharmacol Sci (2013) 2013:308249. 10.1155/2013/308249 24382957 PMC3872021

[51] Ahmad Z . The uses and properties of almond oil. Complement Ther Clin Pract (2010) 16(1):10–2. 10.1016/j.ctcp.2009.06.015 20129403

[52] Boskabady MH Shafei MN Saberi Z Amini S . Pharmacological effects of *Rosa damascena* . Iran J Basic Med Sci (2011) 14(4):295–307. 23493250 PMC3586833

[53] Zhang Y Ning Z Lu C Zhao S Wang J Liu B Triterpenoid resinous metabolites from the genus boswellia: pharmacological activities and potential species-identifying properties. Chem Cent J (2013) 7(1):153. 10.1186/1752-153X-7-153 24028654 PMC3847453

[54] Siddiqui MZ . *Boswellia serrata*, a potential antiinflammatory agent: an overview. Indian J Pharm Sci (2011) 73(3):255–61. 10.4103/0250-474X.93507 22457547 PMC3309643

[55] Almeida-da-Silva CLC Sivakumar N Asadi H Chang-Chien A Qoronfleh MW Ojcius DM Effects of frankincense compounds on infection, inflammation, and oral health. Molecules (2022) 27(13):4174. 10.3390/molecules27134174 35807419 PMC9268443

[56] Cao B Wei XC Xu XR Zhang HZ Luo CH Feng B Seeing the unseen of the combination of two natural resins, frankincense and myrrh: changes in chemical constituents and pharmacological activities. Molecules (2019) 24(17):3076. 10.3390/molecules24173076 31450584 PMC6749531

[57] Borotová P Čmiková N Galovičová L Vukovic NL Vukic MD Tvrdá E Antioxidant, antimicrobial, and anti-insect properties of *Boswellia carterii* essential oil for food preservation improvement. Horticulturae (2023) 9(3):333. 10.3390/horticulturae9030333

[58] Mohammadi A Arabshahi-Delouee S Zinoviadou K Galanakis C . Antioxidant properties of various solvent extracts of Indian frankincense (*Boswellia serrata*) oleogum resin. Iranian Food Sci Technol Res J (2017) 13(3):28–38. 10.22067/ifstrj.v1396i3.61520

[59] Popko K Gorska E Stelmaszczyk-Emmel A Plywaczewski R Stoklosa A Gorecka D Proinflammatory cytokines Il-6 and TNF-α and the development of inflammation in obese subjects. Eur J Med Res (2010) 15(Suppl. 2):120–2. 10.1186/2047-783x-15-s2-120 21147638 PMC4360270

[60] Rajkumar M Presley SID Govindaraj P Girigoswami K Meenambigai K Deepak P Biomedical prowess of Clitoria mariana (L.) flower methanolic extract: a comprehensive *in vitro*, *Ex Vivo*, and *in vivo* evaluation. Chem Biodivers (2025) 22(12):e01051. 10.1002/cbdv.202501051 40773726

